# Supplementary material for: Ionomer-free and recyclable porous-transport electrode for high-performing proton-exchange-membrane water electrolysis
Source: Nat Commun. 2023 Jul 31;14:4592. doi: 10.1038/s41467-023-40375-x (PMC10390546; doi:10.1038/s41467-023-40375-x)
Supplement: Supplementary file 1 — Supplementary Information [file 41467_2023_40375_MOESM1_ESM.docx]

**Supplementary Information**

**Ionomer-free and recyclable porous transport electrode for high-performing proton exchange membrane water electrolysis**

*Lee et al.*

**
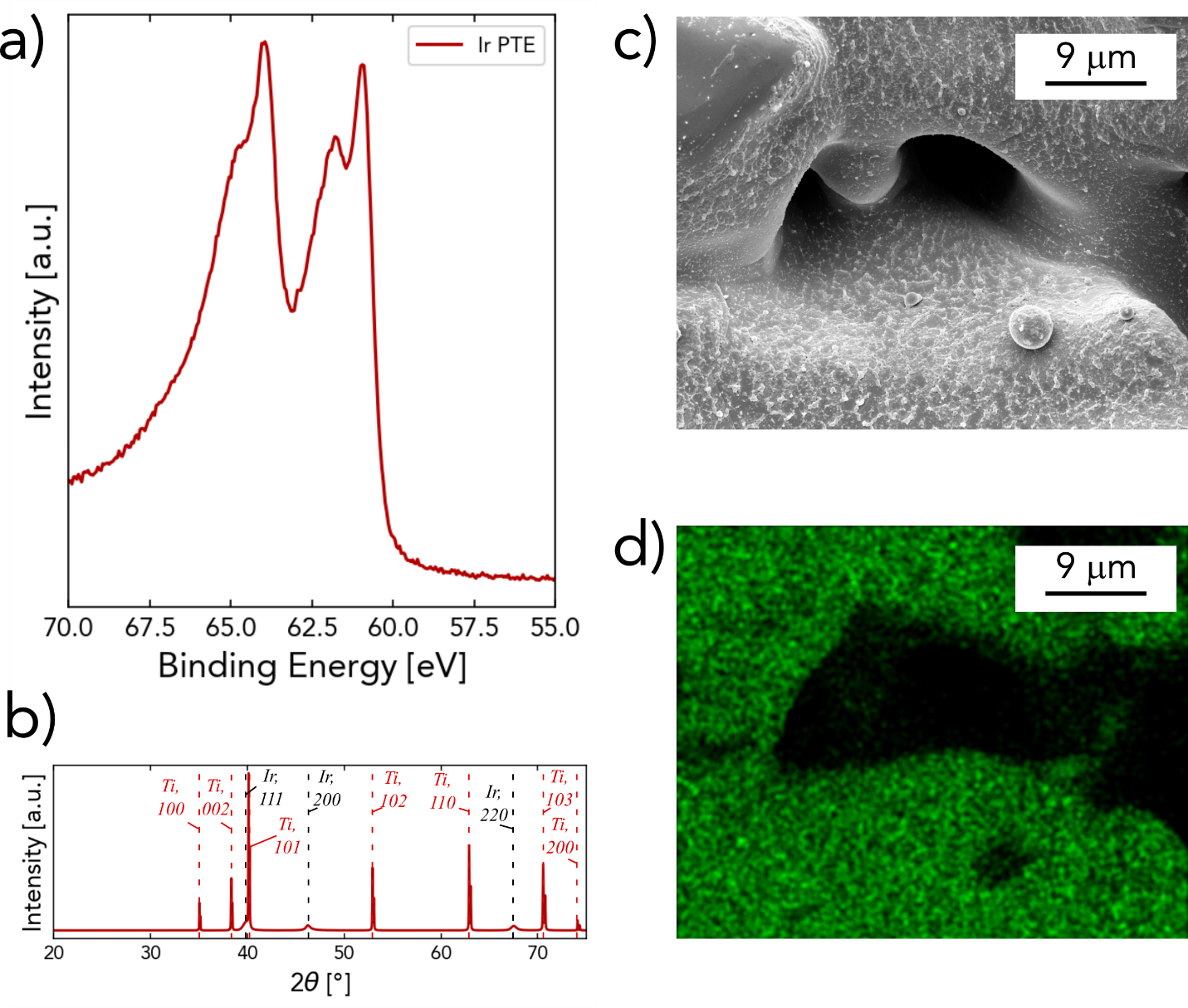
**

**Supplementary Figure 1: Physical characterization of Ir PTE**. **a** XPS and **b** XRD measurements of the Ir PTE fabricated in this study. **c** Ir coating on the PTE can be seen at higher magnification SEM and **d** EDS images.


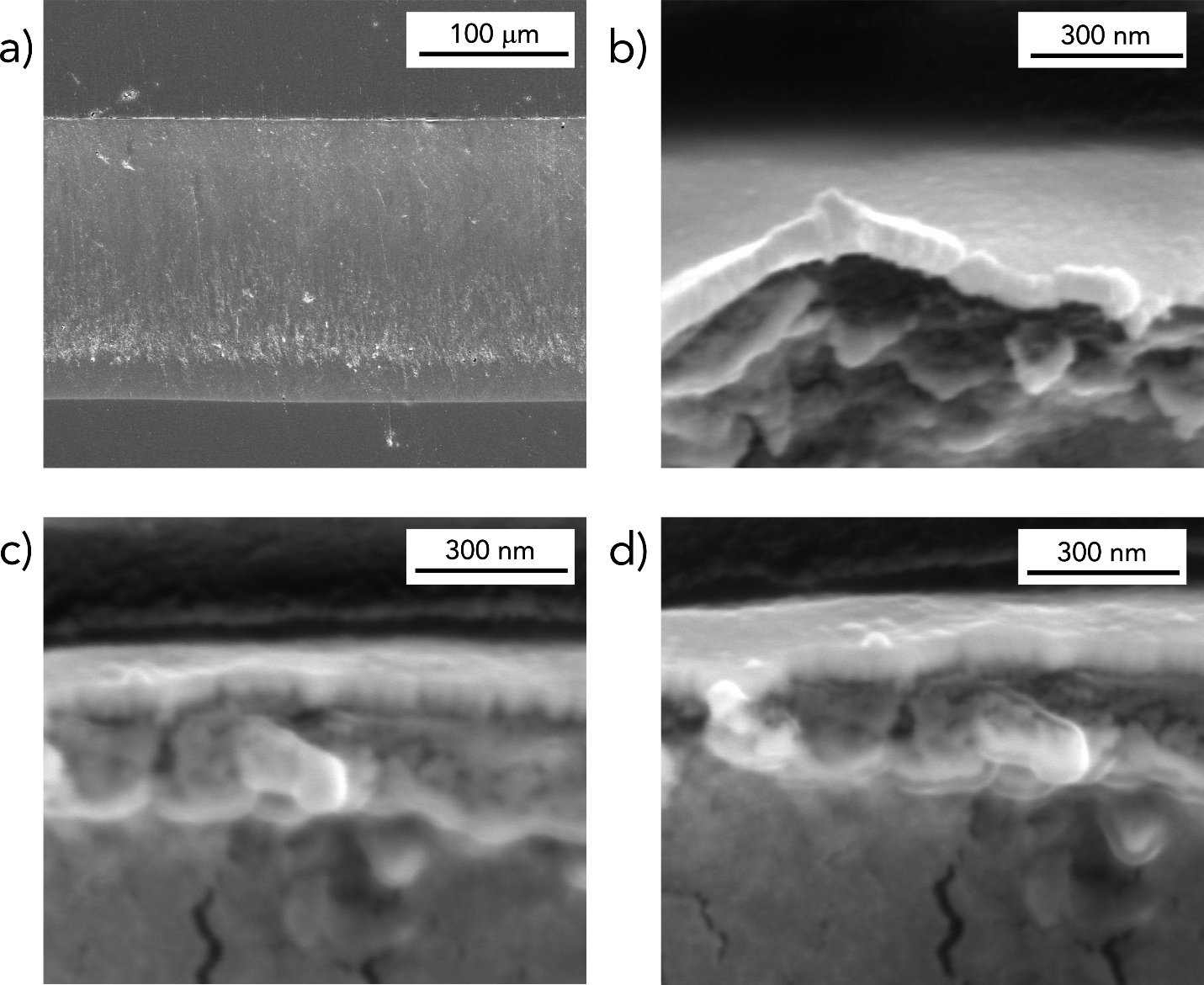


**Supplementary Figure 2:** A cross sectional image of the Ir coating. Ir coating at a loading of 0.085 mg_Ir_/cm^2^ was applied to Nafion 117 to measure the Ir coating thickness using SEM. **a** low magnification image of the Ir coating. **b, c, d**, high magnification of the Ir coating. The Ir coating has thicknesses between roughly 90 to 100 nm and is homogeneous across coated surface.

**Supplementary Discussion 1**

**Tailoring the surface compositions of Ir PTEs**

To finetune the Ir PTE surface composition and understand its potential impact on PEMWE performance, we conducted a post thermal treatment in air from 200 to 500˚C. As sintering temperature increases, there is an increase of iridium oxide in the PTE as indicated by XPS measurements (Supplementary Figure 3a). However, XRD only shows the presence of metallic Ir, implying either very thin or amorphous oxide formation after thermal annealing (Supplementary Figure 3b). XRD measurements also show increases in Ir crystallinity as more existence of diffraction peaks of (200) and (220) at higher temperatures (JCPDS 06-0598). The electrolyzer performance of the sintered Ir PTEs are as shown in Supplementary Figure 3c. The change in performance with sintering temperature is subtle up to 400˚C, even with increased content of iridium oxide in the catalyst layer. From Tafel plots (Supplementary Figure 3d), the measured Tafel slopes (Supplementary Figure 3d) increase with increased oxide in iridium. This is likely due to the decrease of metallic Ir content and increase of oxide content, which is shown to be less active for oxygen evolution reaction.^1,2^For the PTE sintered at 500˚C, a very thick oxide layer formed on the titanium phase in the PTL, which significantly decreased the electronic conductivity and translated to high ohmic loss in the polarization curve. A hint of blue oxide layer was seen on the back side of the PTL sintered at 500˚C (Supplementary Figure 4).


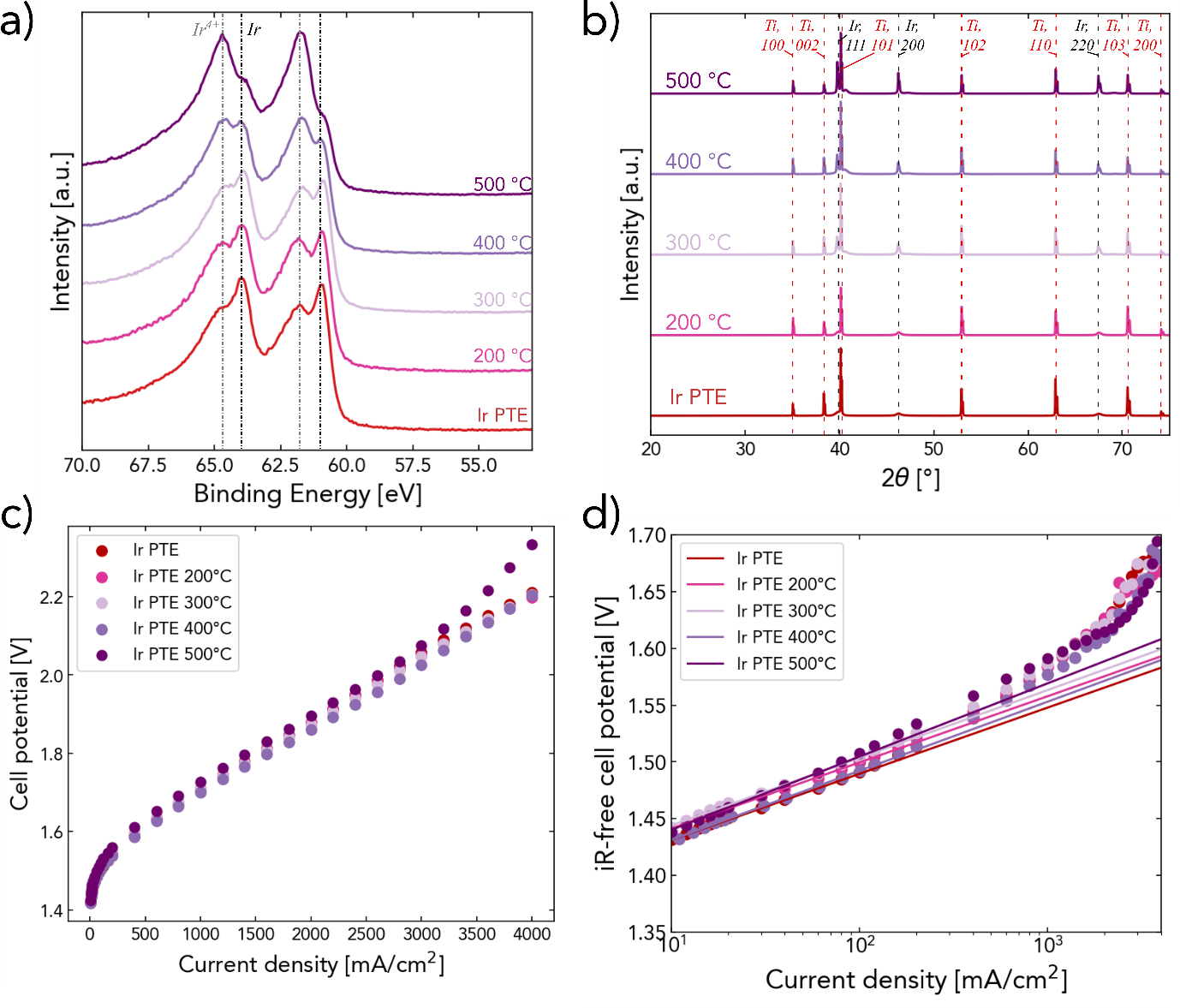


**Supplementary Figure 3**: **The impact of post thermal annealing temperature on the Ir PTE.** **a** XPS and **b** XRD patterns. **c** polarization curves and **d** Tafel slope measurements from Ir PTEs (58.3, 60.4, 62.9, 62.2, 64.5 mV/dec for PTEs processed at room temperature, 200, 300, 400, and 500˚C, respectively). Higher contents of oxides present for Ir PTEs processed at higher temperature.


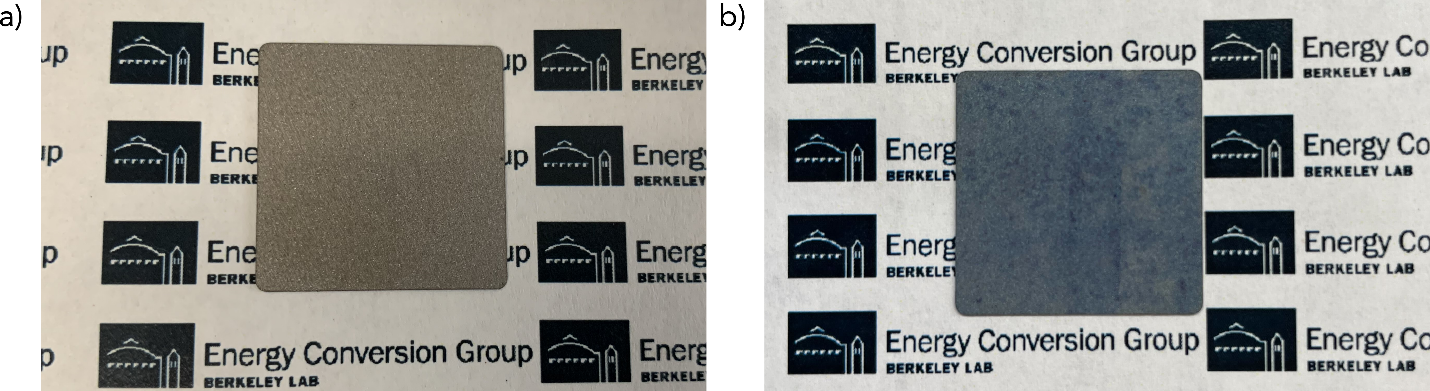


**Supplementary Figure 4: The images show oxide formation on the Ir PTE with post processing at higher temperature.** The back side of **a** Ir PTE and **b** Ir PTE post processed at 500 ˚C. Blue layer over titanium phase seen in **b,** suggest formation of thick oxide layer during the post processing. Ir PTEs are 5 cm^2^ in area.


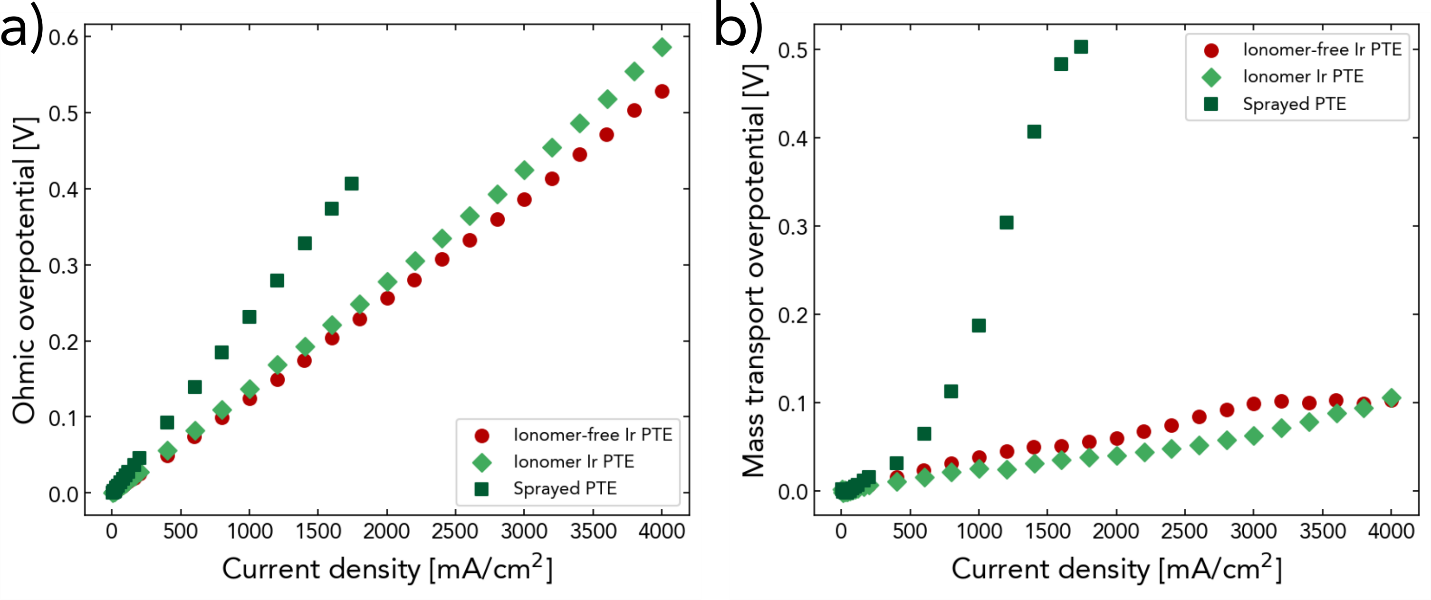


**Supplementary Figure 5: Comparing cell performance from electrolyzers assembled with the Ir PTE, Nafion ionomer-coated PTE, and conventional ultrasonic spray coated Ir/Nafion PTE.** **a** ohmic and **b** mass transport overpotentials are shown for each PTEs. Traditional ultrasonic sprayed PTE experiences undesirably high ohmic and mass transport overpotentials.

**
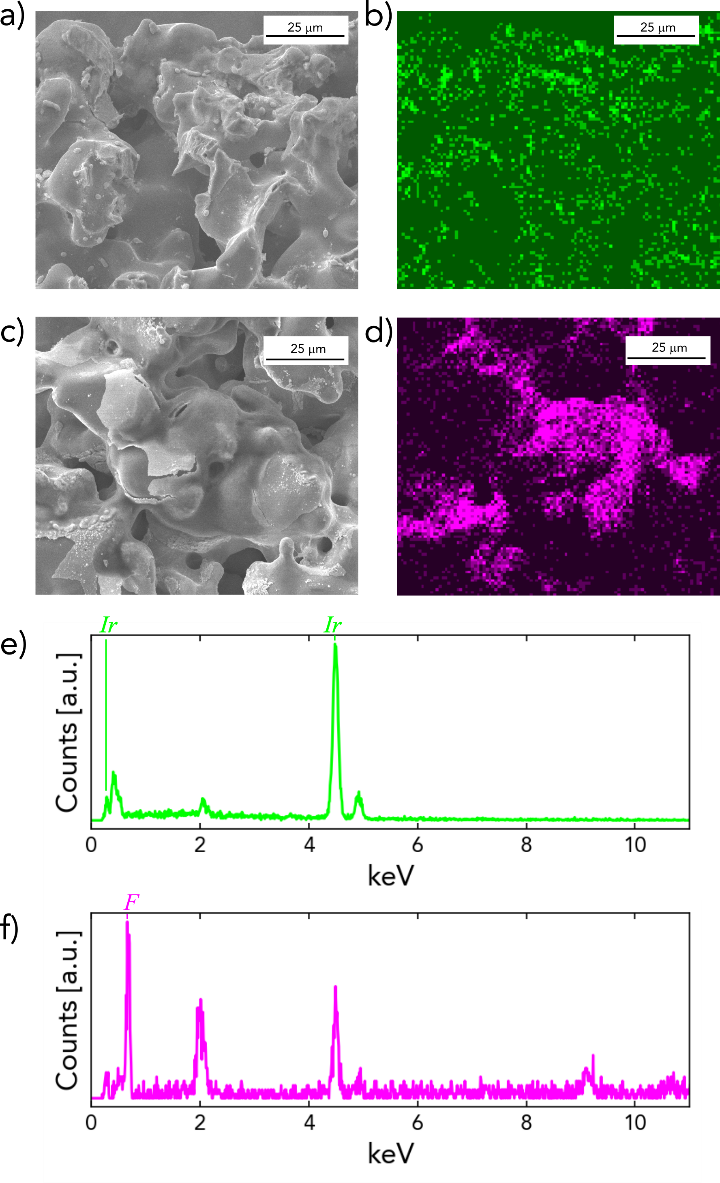
**

**Supplementary Figure 6: The cross-sectional image of the ultrasonic spray coated porous transport electrode (PTE) and surface SEM image of ionomer coated Ir PTE.** **a** Penetration of catalysts observed from cross sectional image of the spray coated PTE. **b** EDS mapping of iridium shows catalyst particles wasted inside PTL. **c** Surface SEM image and **d** EDS mapping of the fluorine in the ionomer coated Ir PTE after PEMWE operation. **e** EDS spectrum of iridium from **b.** **f** EDS spectrum of fluorine from **d**.

**
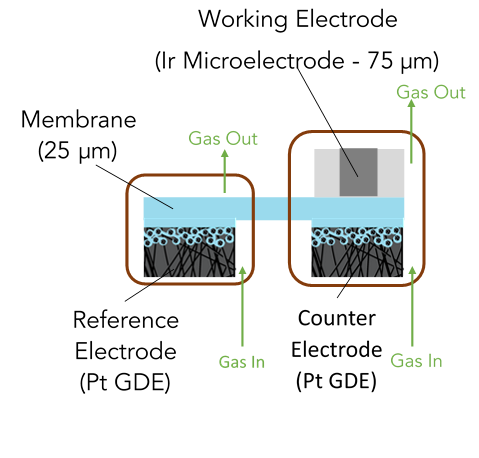
**

**Supplementary Figure 7: A schematic of the microelectrode setup.** The working electrode is the Ir microelectrode, which is in contact with the membrane. Pt GDE is used as both counter and reference electrodes.

**
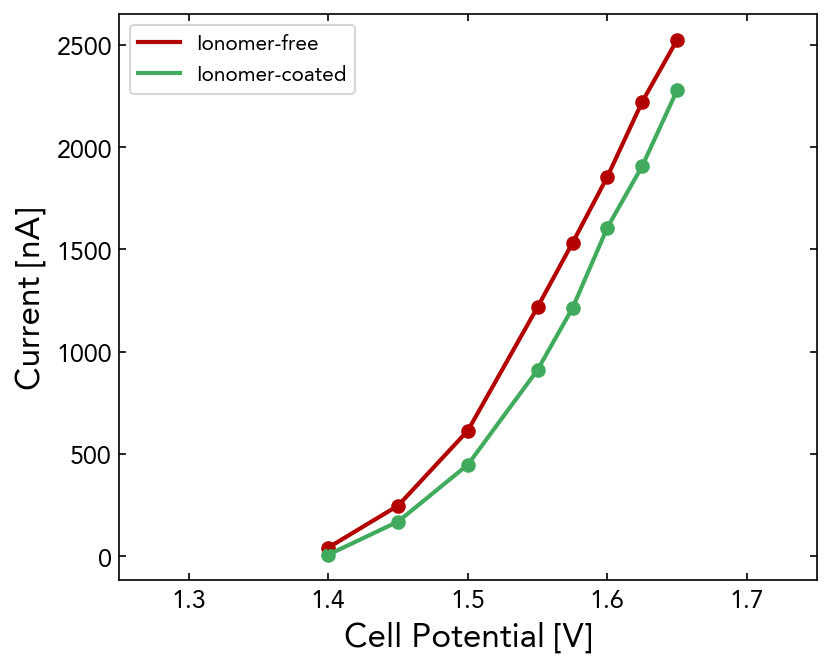
**

**Supplementary Figure 8: A polarization curve measured with microelectrode, in absolute currents.** Small active area (75 µm diameter) leads to very low current flow, in nA scale, and therefore provides insights on kinetics. The OER kinetics without ionomer demonstrate higher performance.


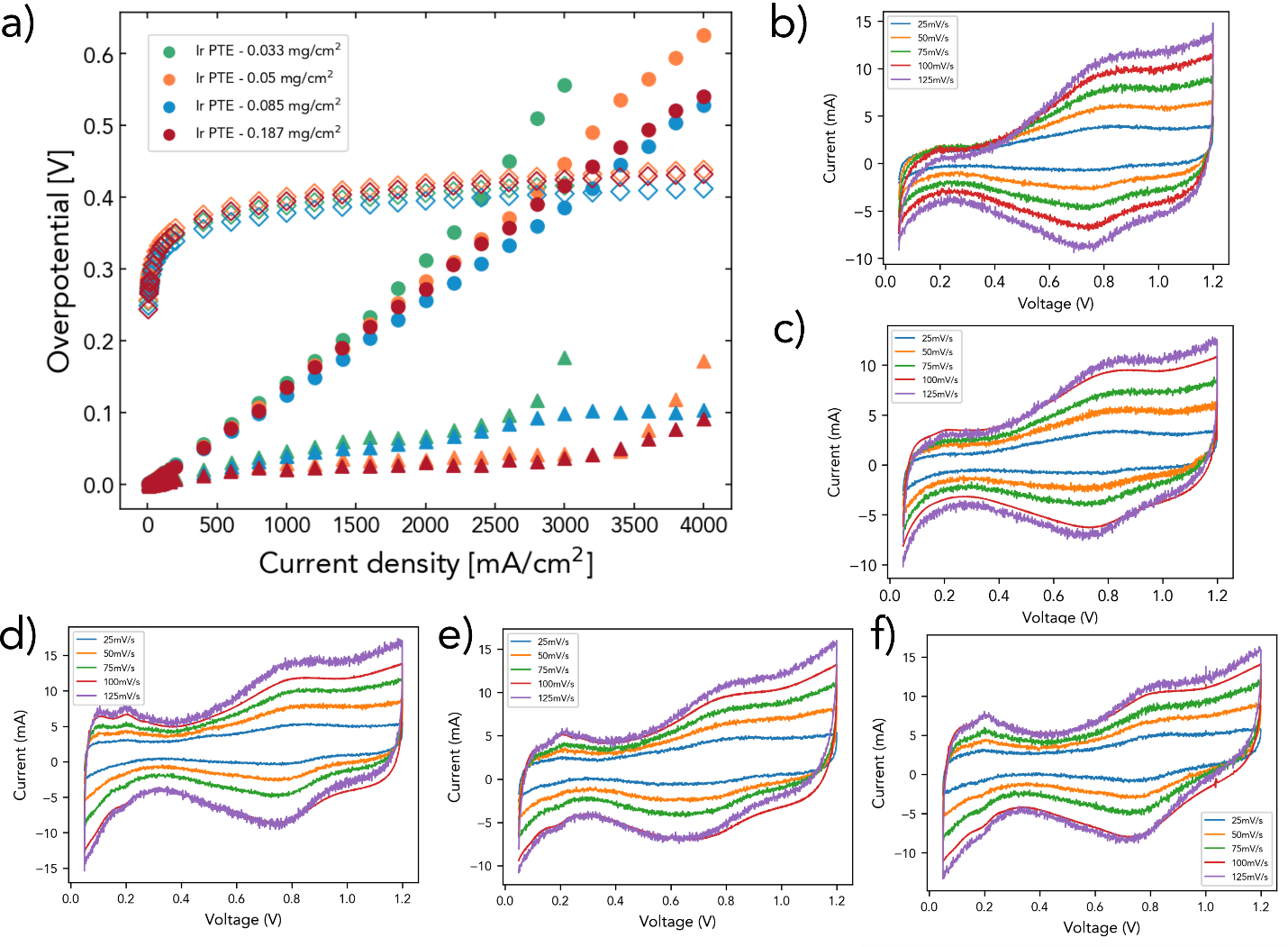


**Supplementary Figure 9: Applied voltage breakdowns and cyclic voltammograms measured from Ir PTEs at lower loadings. a** applied voltage breakdown at various Ir loadings. Loadings impact ohmic and kinetics overpotentials throughout current densities, while mass transport exacerbates at high current densities, near limiting currents. Measured cyclic voltammograms for **b** 0.033 mg_Ir_/cm^2^. **c** 0.05 mg_Ir_/cm^2^. **d** 0.085 mg_Ir_/cm^2^. **e** 0.187 mg_Ir_/cm^2^ and **f** laser ablated PTE at 0.085 mg_Ir_/cm^2^.


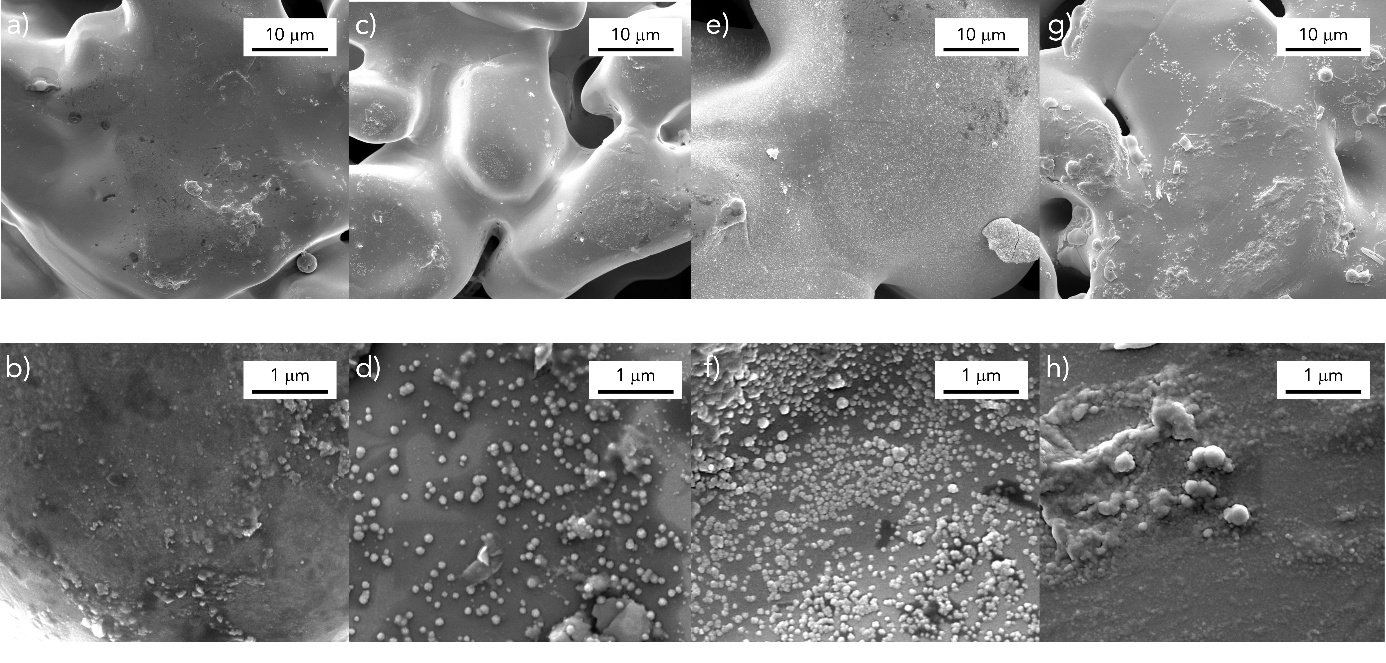


**Supplementary Figure 10: Morphological difference for Ir PTE at various Ir loadings.** SEM images of the Ir PTEs at loadings of **a,** **b:** 0.033, **c, d:** 0.05, **e, f:** 0.085 and **g, h:** 0.187 mg_Ir_/cm^2^. Ir layer becomes denser as the loading increases.


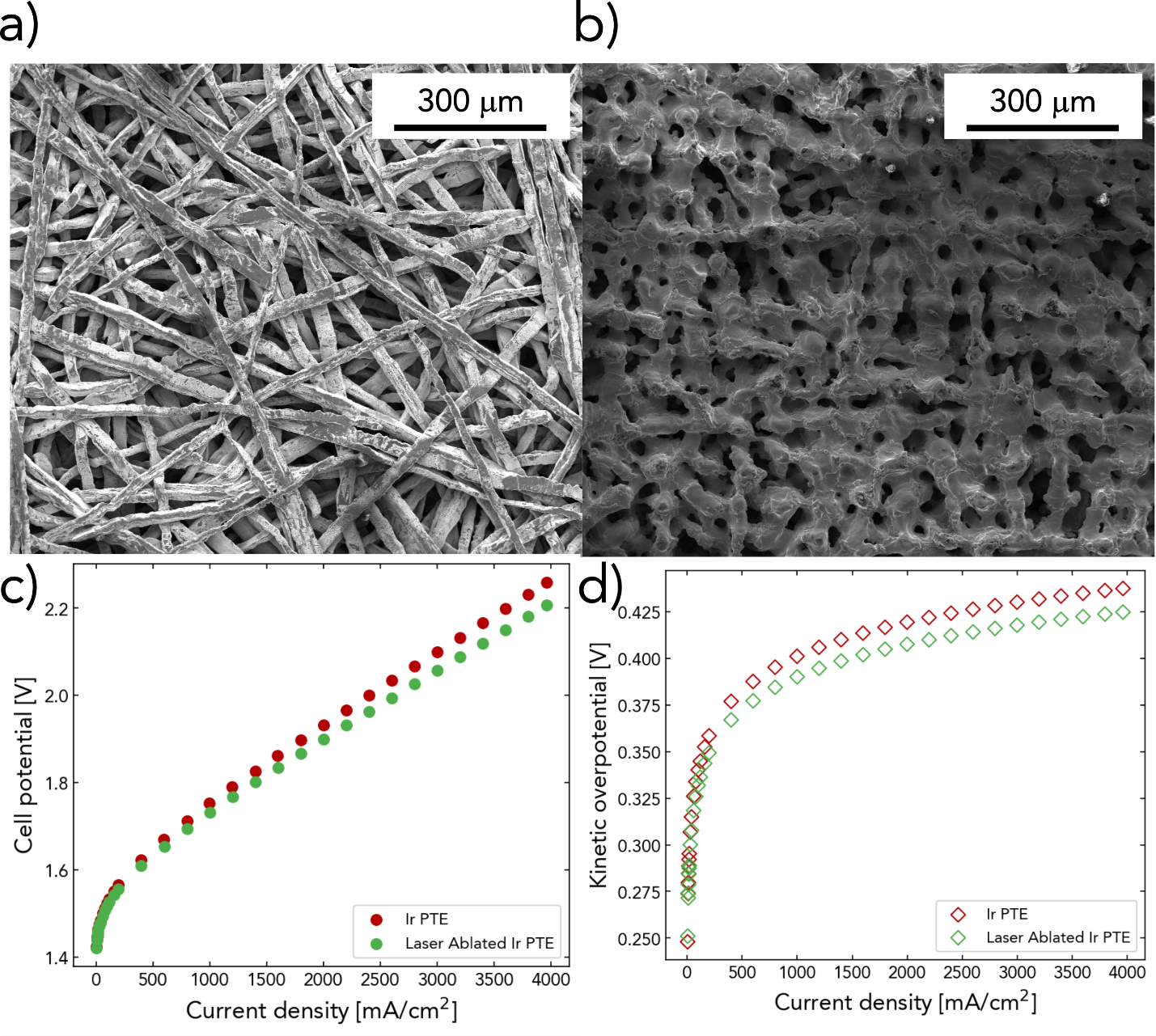


**Supplementary Figure 11: Impact of laser ablation on the fiber-based Ir PTE.** SEM images of **a** fiber-based PTL and **b** laser ablated fiber-based PTL. The electrochemical performance compassion of **c** polarization curves and **d** kinetic overpotential between fiber-based Ir PTE and laser ablated fiber-based Ir PTE. Laser ablation significantly enhances Ir PTE performance through enhancing electrode kinetics.


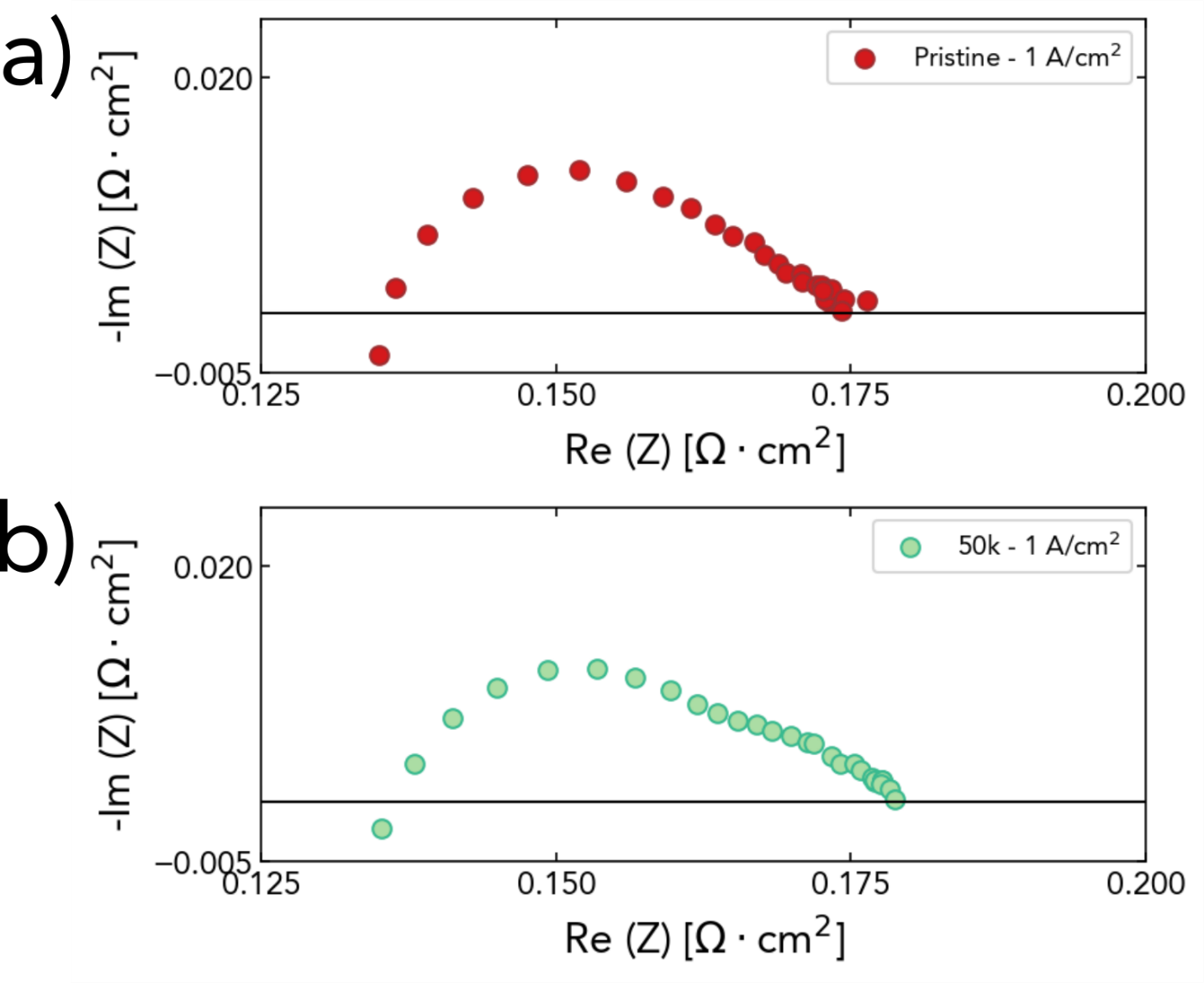


**Supplementary Figure 12:** **Durability of Ir PTE analyzed from electrochemical impedance spectroscopy measurements**. The EIS comparison of **a** before accelerated stress test (AST). **b** after 50k of ASTs. Electrochemical impedance spectroscopy measurements are acquired at 1 A/cm^2^ during AST cycles.

**
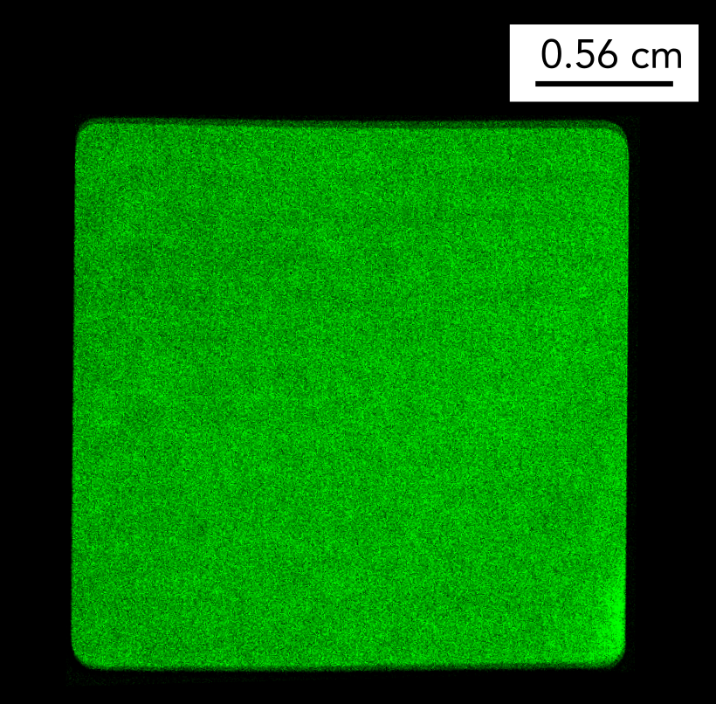
**

**Supplementary Figure 13: X-ray fluorescence (XRF) mapping of the Ir from the Ir PTE after 50k potential cycles.** Ir catalysts remain uniformly distributed even after AST cycles.


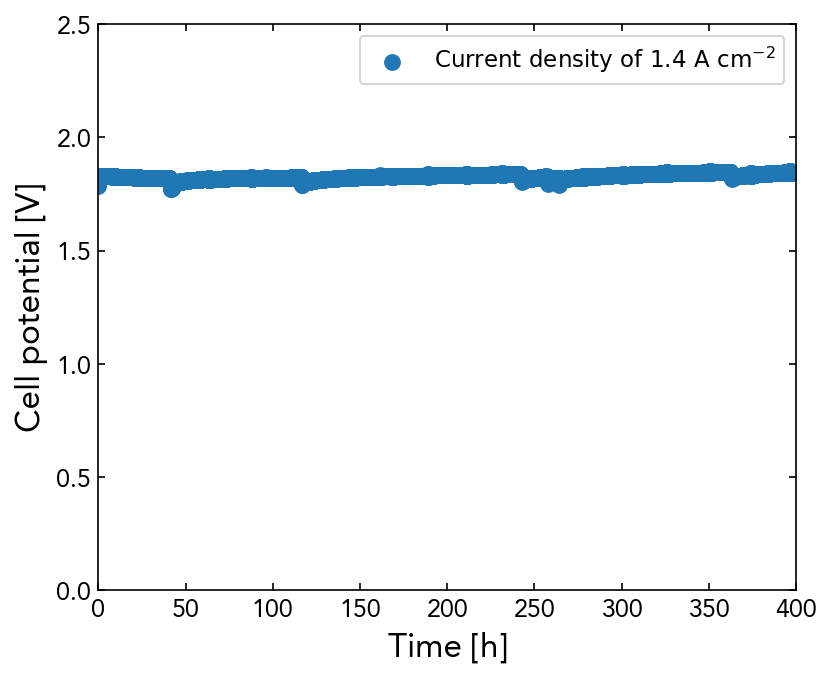


**Supplementary Figure 14:** Short-term durability at constant current hold of 1.4 A/cm^2^ using Ir PTE at anode catalyst loading of 0.085 mg_Ir_/cm^2^ and cathode loading of 0.1 mg_Pt_/cm^2^. Membrane: Nafion 117, cell temperature: 80 ℃. Average degradation rate is 45 µV/hour. Fresh water had to be replenished, which led to water tank temperature fluctuation and small voltage oscillation.


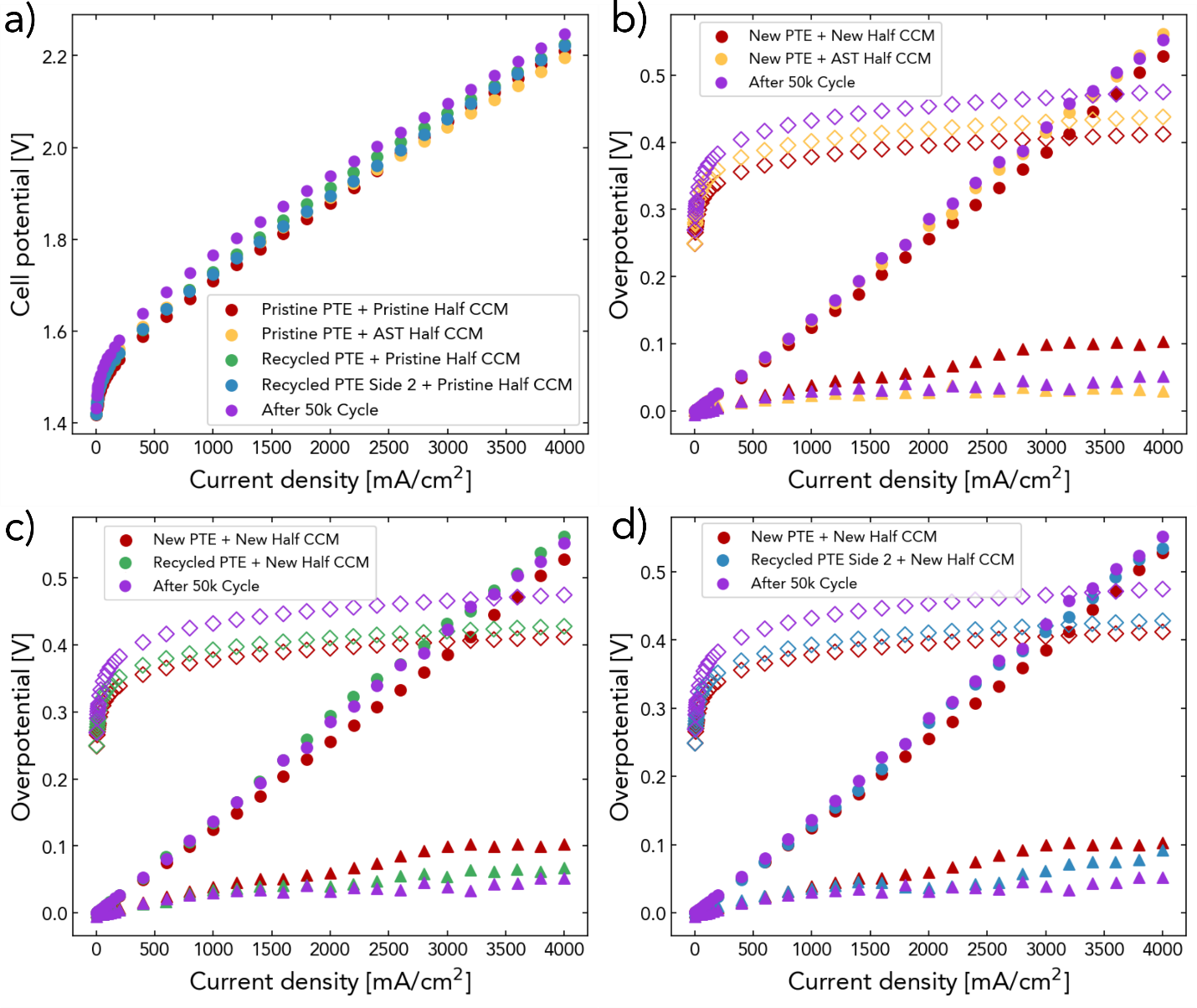


**Supplementary Figure 15: Direct comparison of electrochemical performance of different recycling scenarios. a** Polarization curves measured for varying scenarios. Overpotential breakdowns for **b** scenario 1. **c** scenario 2. **d** scenario 3. Performance rebounds back near pristine with recycled catalysts.


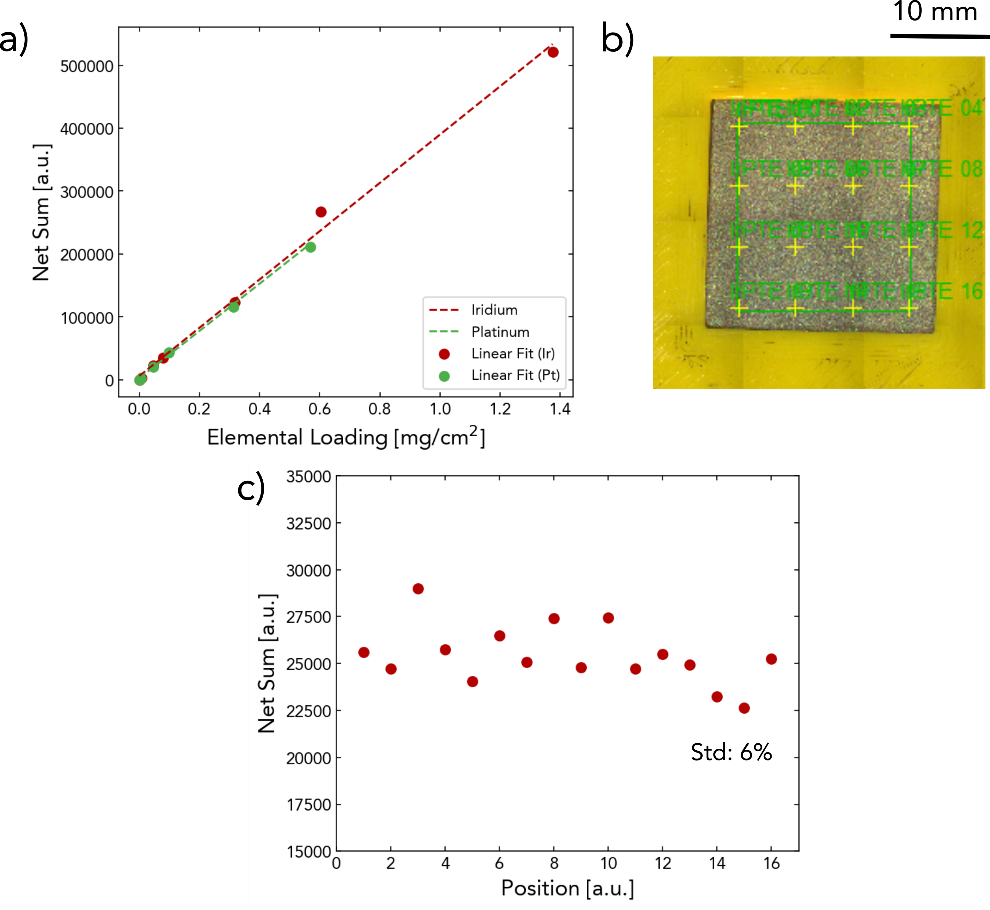


**Supplementary Figure 16: X-ray fluorescence calibration curves and a sample data set for measuring Ir and Pt loadings.** The XRF used to measure anode Ir and cathode Pt were calibrated using measurements from Ir and Pt standards as well as with a 0 mg/cm2 blank. **a** Calibration curves obtained using standards. **b** example of Ir PTE being measured with an XRF. **c** example of XRF measurement data, where measured Ir loading was 0.052 mg_Ir_/cm^2^ with standard deviation of 6%.

**Supplementary Table 1.** **PTE performance evaluation with literature**. The comparison was conducted at identical operating conditions of 80 ˚C and N117 membrane with a PTE configuration. Our presented work achieves 28-fold reduction in loading while reducing cell potential by 33 mV at 1.8 A/cm^2^.

| Anode Loading  (mg_Ir_/cm^2^) | Cathode Loading  (mg_Pt_/cm^2^) | Temperature (℃) | Membrane | Configuration | Potential @ 1.8 A/cm^2^ [V] | reference |
| --- | --- | --- | --- | --- | --- | --- |
| 0.085 | 0.1 | 80 | N117 | PTE | 1.84 | This Work |
| 1.4 | 0.5 | 80 | N117 | PTE | 1.98 | ^3^ |
| 2.0 | 2.0 | 80 | N117 | PTE | 1.87 | ^4^ |
| 0.1 | 0.4 | 90 | N212 | PTE | 1.77 | ^5^ |
| 2.5 | 0.95 | 90 | N115 | CCM | 1.94 | ^6^ |
| 2.0 | - | 80 | N115 | CCM | 1.85 | ^7^ |
| 1.5 | 0.5 | 80 | N117 | CCM | 1.99 | ^8^ |
| 0.085 | 0.38 | 55 | N117 | CCM | 1.97 | ^9^ |

**References**

1. Danilovic, N. *et al.* Activity−Stability Trends for the Oxygen Evolution Reaction on Monometallic Oxides in Acidic Environments. *J. Phys. Chem. Lett.* **5**, 2474-2478 (2014).

2. Kim, Y. T. *et al.* Balancing activity, stability and conductivity of nanoporous core-shell iridium/iridium oxide oxygen evolution catalysts. *Nat. Commun.* **8**, 1–8 (2017).

3. Bühler, M., Holzapfel, P., McLaughlin, D. & Thiele, S. From Catalyst Coated Membranes to Porous Transport Electrode Based Configurations in PEM Water Electrolyzers. *J. Electrochem. Soc.* **166**, F1070–F1078 (2019).

4. Pushkarev, A. S. *et al.* On the influence of porous transport layers parameters on the performances of polymer electrolyte membrane water electrolysis cells. *Electrochim. Acta* **399**, 139436 (2021).

5. Lee, B. S. *et al.* Polarization characteristics of a low catalyst loading PEM water electrolyzer operating at elevated temperature. *J. Power Sources* **309**, 127–134 (2016).

6. Stiber, S. *et al.* Porous Transport Layers for Proton Exchange Membrane Electrolysis Under Extreme Conditions of Current Density, Temperature, and Pressure. *Adv. Energy Mater.* **11**, 2100630 (2021).

7. Schuler, T. *et al.* Hierarchically Structured Porous Transport Layers for Polymer Electrolyte Water Electrolysis. *Adv. Energy Mater.* **10**, 1903216 (2020).

8. Pham, C. Van, Escalera-López, D., Mayrhofer, K., Cherevko, S. & Thiele, S. Essentials of High Performance Water Electrolyzers – From Catalyst Layer Materials to Electrode Engineering. *Adv. Energy Mater.* **11**, 2101998 (2021).

9. Lopata, J. *et al.* Effects of the Transport/Catalyst Layer Interface and Catalyst Loading on Mass and Charge Transport Phenomena in Polymer Electrolyte Membrane Water Electrolysis Devices. *J. Electrochem. Soc.* **167**, 064507 (2020).
